# New Efficient Visible-Light-Driven Photocatalyst of Chitin-Modified Titanium Dioxide/Carbon Fiber Composites for Wastewater

**DOI:** 10.1038/s41598-019-52833-y

**Published:** 2019-11-08

**Authors:** Kui Mao, Xiaowen Wu, Xin Min, Zhaohui Huang, Yan-gai Liu, Minghao Fang

**Affiliations:** Beijing Key Laboratory of Materials Utilization of Nonmetallic Minerals and Solid Wastes, National Laboratory of Mineral Materials, School of Materials Science and Technology, China University of Geosciences (Beijing), 29 Xueyuan Road, Beijing, 100083 China

**Keywords:** Materials chemistry, Materials chemistry, Materials chemistry, Photocatalysis, Photocatalysis

## Abstract

To improve the catalyst properties of TiO_2_ under visible light irradiation, chitin-modified TiO_2_ was synthesized via a hydrothermal method on the surface of carbon fibers. The microstructure and interface properties of the so-prepared photocatalyst were investigated via X-ray diffraction, scanning electron microscopy, X-ray photoelectron spectroscopy, and UV-visible diffuse reflectance spectroscopy. Our results indicated that the synergetic effect of the crystal phase of TiO_2_, carbon fiber, and chitin is the main reason leading to the improvement of the photocatalytic activity of the composite catalyst. The modified TiO_2_ sample with chitin content of 0.6 wt% exhibited the highest photocatalytic activity under visible light irradiation when RhB was chosen as the target degradation product. Compared to the pure TiO_2_/carbon fiber, the sample of TiO_2_/carbon fiber with 0.6 wt% of chitin exhibits enhanced visible light activity with an apparent rate of degradation about 2.25 times. The enhancement of the photocatalytic performance of the sample with chitin can be attributed to the relatively high adsorption capacity of the particular network structure and photosensitivity of chitin, which can effectively separate the photoelectron-hole pair recombination. Furthermore, the new composite photocatalyst shows excellent catalytic stability after multiple degradation cycles, indicating that it is a promising photocatalytic material for degrading organic pollutants in wastewater.

## Introduction

With the economic development and population growth, the environmental pollution caused by organic matter is becoming more and more serious. The clean water that human beings depend on is gradually reducing. Thus, the environment cleaning or wastewater treatment has gained increasing attention^1–3^. Various technologies have been developed in the field of wastewater treatment, such as coprecipitation^4^, adsorption^5^, electrochemistry^6^, and membrane dialysis^7^. Unfortunately, although these methods have been applied to a varying extent, they still present several disadvantages. For instance, the coprecipitation method requires high cost^8^. The adsorption method does not completely treat the wastewater and cannot effectively convert the toxic substances into other non-toxic substances^9^. The electrochemical method leads to the formation of intermediate products with strong oxidative power towards the contaminant^10^. Finally, the membrane permeation is only suitable for small organic molecules and inorganic ions with poor selectivity. Although the operating conditions are straightforward, the efficiency is low with multiple factors affected (such as pH, electrolyte, and electrode material)^11^. The photocatalytic technology has been widely researched in recent years, as it is one of the most environmentally friendly wastewater treatment methods because of its mild reaction conditions, low energy consumption, no formation of secondary pollutants, simple operation, and good selectivity^12–15^. Among many semiconductor materials, TiO_2_ is considered one of the most promising semiconductors photocatalytic for wastewater treatment and air purification due to its non-toxicity, high stability, low cost, and excellent photocatalytic activity^16–18^. However, owing to the high recombination rate of electron-hole pairs and the wide band gap, the ineffective response of TiO_2_ to visible light significantly limits its application^19–21^. Furthermore, although TiO_2_ has high photocatalytic activity, it still has a significant disadvantage that affects the large-scale industrial application of TiO_2_^22^. TiO_2_ powders are difficult to separate and recover from water, causing secondary pollution^23,24^. To tackle the abovementioned issues, many researchers have focused on loading TiO_2_ powder onto a matrix material^25,26^. For example, Guo *et al*. directly grew TiO_2_ nanosheets arrays on carbon fibers^27^. Long, M. *et al*. successfully used the sol-gel method to prepare cotton/TiO_2_ composites with high photocatalytic activity and stability^28^. In addition, TiO_2_ nanoparticles have been incorporated into an organic matrix to form an organic-inorganic heterojunction photocatalytic composite^29^. For example, Dassanayake, R. S. *et al*. successfully synthesized aerochitin-titania (TiO_2_) composites, which showed high selectivity and multiple utilization performance^30^. Although carbon fiber and cotton have been demonstrated to be used as substrates for growing TiO_2_ nanoparticles, it is unfortunate that TiO_2_ nanoparticles are susceptible to agglomeration on substrates and have a poor synergistic effect, which limits their potential as catalyst supports. Although being an effective modifier due to its special network structure and excellent adsorption and being considered the second most abundant organic polymer on earth after cellulose, chitin has received little if not absent attention^31–33^. The structure of chitin is equivalent to that of cellulose polysaccharide with the hydroxyl functional group of C-2 substituted by the acetamido one, which has a high percentage of nitrogen (5–8%) and outstanding adsorption properties^34^.

In this work, a method based on the coupling of TiO_2_ and chitin was reported for preparing chitin-modified TiO_2_/Carbon fiber composites. Owing to the good bioadsorption, selectivity, and recyclability of chitin, this work aimed to improve the dispersion of TiO_2_ on the carbon fiber surface and to fully exert the synergistic effect of TiO_2_ with chitin and the carbon fiber. Indeed, the so-modified TiO_2_/carbon fiber composites exhibit unique characteristics, such as recycling, recyclability, complete degradation and mineralization of organic matter. These modified TiO_2_/Carbon fiber composites material can efficiently treat the organic matter in wastewater, thus providing a feasible solution for industrialized high-efficiency wastewater treatment.

## Results and Discussion

**Figure 1:**
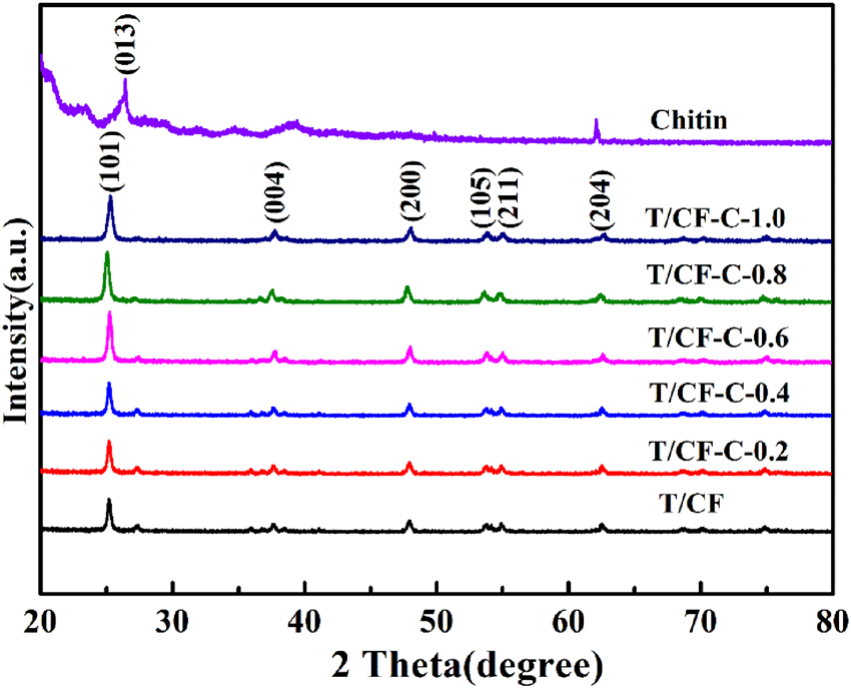
XRD patterns of chitin, T/CF, and the prepared composites.

The phases of chitin, T/CF, and the chitin-modified T/CF composites were characterized via XRD. As shown in Fig. 1, the prepared T/CF composites exhibit a representative diffraction peak of TiO_2_. There is no obvious diffraction peak of chitin: this may result from the hydrogen bond between TiO_2_ and chitin that interferes with the crystallization of chitin, indicating that TiO_2_ is completely embedded in the matrix of chitin as the inorganic phase. The characteristic peaks of TiO_2_ are at 25.2°, 37.7°, 48.1°, 53.9°, 55.1°, and 62.8°, corresponding to the (101), (004), (200), (105), (211), and (204) crystal faces of anatase TiO_2_ (JCPDS 21–1272) as they appear in the so-prepared photocatalyst^35^. Hence, the prepared photocatalytic material exhibits a high photocatalytic activity.

**Figure 2:**
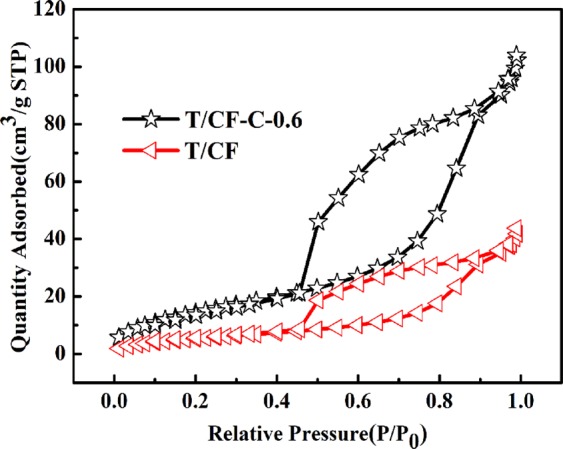
N_2_ adsorption and desorption isotherms of T/CF and T/CF-C-0.6.

The pore structure of the prepared T/CF composites was further investigated by BET. The typical results BET data are shown in Table 1, where it can be seen that the addition of chitin significantly changed the specific surface area of the T/CF composites. The results show that T/CF-C-0.6 sample has a specific surface area of 73.3591 m^2^/g, which is 2.5 times higher than that of the unadded modifier of T/CF, resulting in enhanced adsorption of the dye on the composites. Since chitin itself is the particular network structure, TiO_2_ nanoparticles fill the chitin network voids to homo-disperse and increase the specific surface area of the sample. The N_2_ adsorption-desorption isotherm of samples of T/CF and T/CF-C-0.6 is shown in Fig. 2. It can be observed that the T/CF and T/CF-C-0.6 samples show a type IV adsorption isotherm with an H_2_ hysteresis loop in the range (P/P_0_) of 0.5–0.9 according to the International Union of Pure and Applied Chemistry (IUPAC), indicating the presence of mesoporous (2–50 nm)^36^. This is beneficial to the multi-layer adsorption of pollutant molecules in aqueous solution.

**Table 1:** Surface and pore volume characterization of T/CF and the prepared composites.

Samples	T/CF	T/CF-C-0.2	T/CF-C-0.4	T/CF-C-0.6	T/CF-C-0.8	T/CF-C-1.0
S_BET_(m^2^/g)	28.3204	31.0856	52.6843	73.3591	33.7821	26.1793
Pore Volume(cm^3^/g)	0.1124	0.1746	0.2461	0.2652	0.1632	0.1298

**Figure 3:**
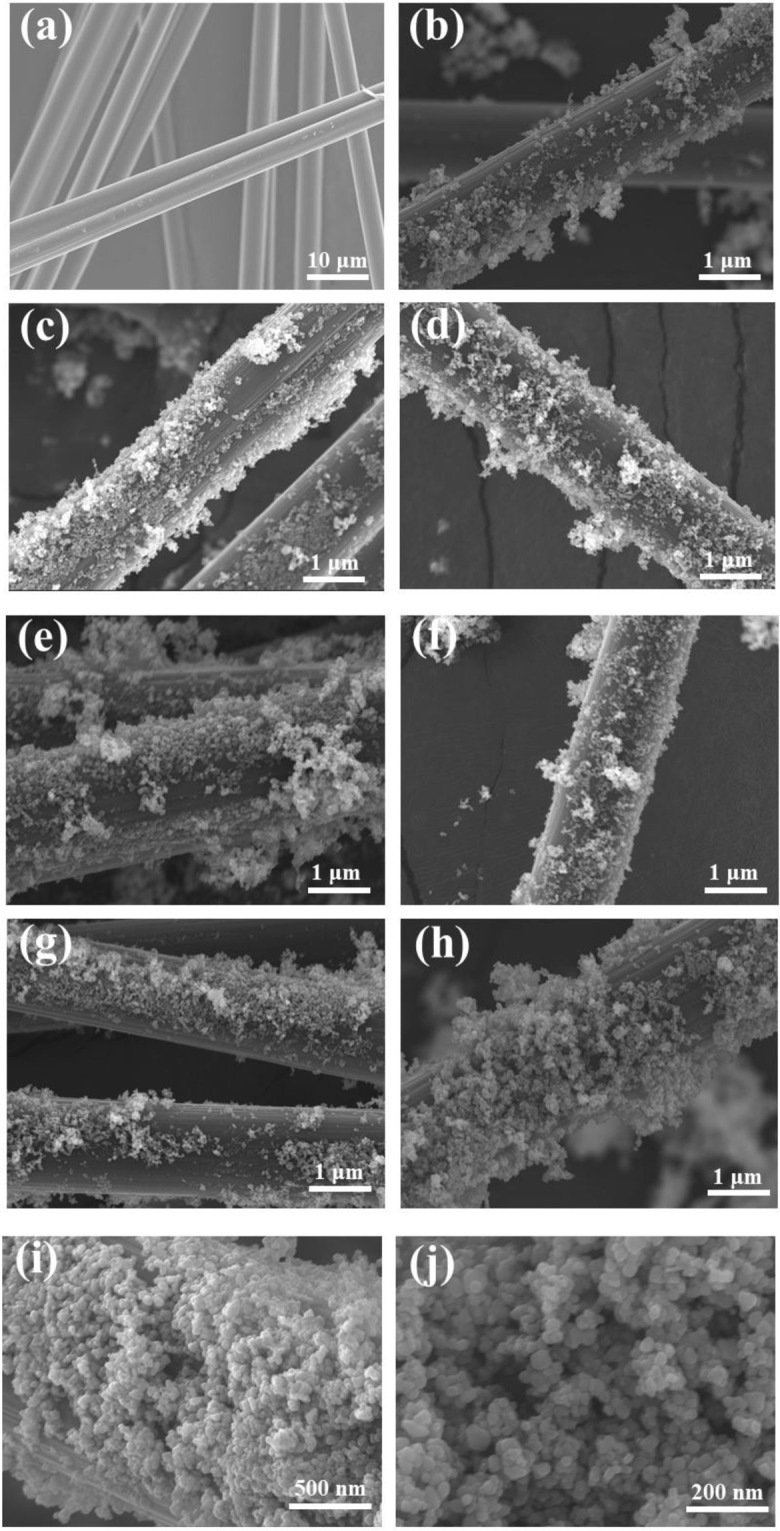
SEM images of carbon fiber (**a**), T/CF, T/CF-C-0.2, T/CF-C-0.4, T/CF-C-0.6, T/CF-C-0.8, and T/CF-C-1.0, respectively (**b**–**g**), and T/CF-C-0.6 (**h**–**j**).

Figure 3 shows the SEM images of the T/CF composites. As can be seen in Fig. 3a, the carbon fiber has a uniform diameter of 10 μm with a smooth surface. Figure 3b–g shows the SEM images of composite samples of T/CF, T/CF-C-0.2, T/CF-C-0.4, T/CF-C-0.6, T/CF-C-0.8, and T/CF-C-1.0, respectively. It can be observed that TiO_2_ nanoparticles have been loaded on the surface of carbon fiber in different extents. Moreover, the SEM image of composites without chitin (Fig. 3b) shows that the TiO_2_ has poor loadability and dispersibility on the surface of carbon fiber. Chitin is a frequently used modifier to disperse the TiO_2_ nanoparticles on the carbon fiber surface more uniformly, reducing the aggregation. It can be shown in Fig. 3e,h,i,j that, when the amount of chitin added is 0.6%, the loadability and dispersibility of TiO_2_ are relatively good, with the distribution of TiO_2_ nanoparticles being significantly more uniform than that of nanoparticles without chitin as a modifier. Due to the change of the zeta potential on the surface of TiO_2_ in the acidic solution, the presence of titanium acetate and the particular network structure of chitin can enhance the loading and dispersion of TiO_2_ nanoparticles on the carbon fiber surface, while providing a ratio of the specific surface area for adsorbing and degrading organic wastewater^37,38^. This is consistent with the results of XRD and BET.

**Figure 4:**
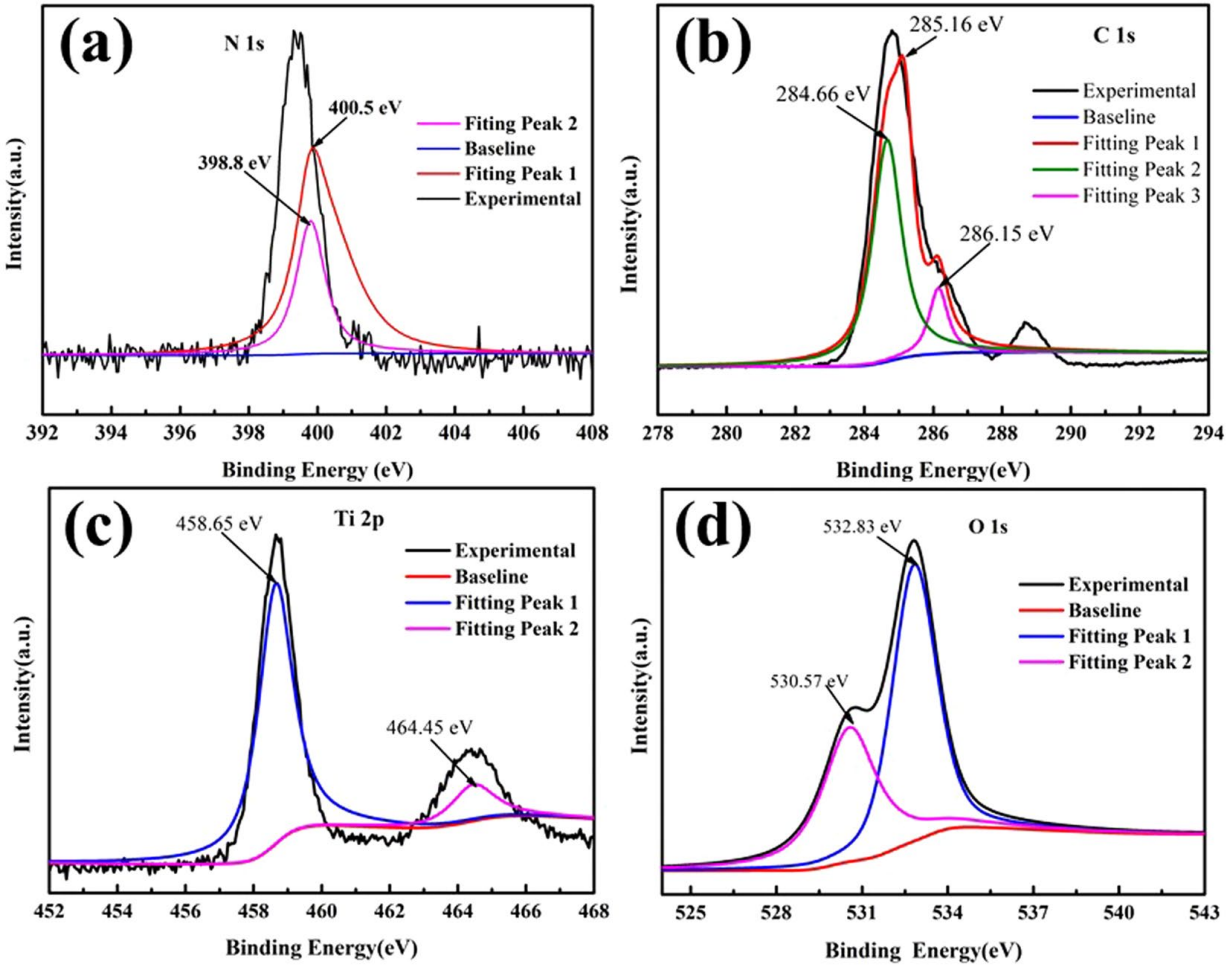
XPS spectra of T/CF-C-0.6 sample N 1 s (**a**), C 1 s (**b**), Ti 2p (**c**), and O 1 s (**d**) electrons in the composites.

To further clarify the surface chemical composition of the sample, we investigated the interfacial behavior between TiO_2_ and carbon fiber via XPS. Figure 4 shows the XPS spectrum of a T/CF-C-0.6 sample. According to the N 1 s XPS spectrum in Fig. 4a, the poor adhesion of anions to TiO_2_ is further revealed. A broadband at about 400 eV can be fitted into two peaks at 398.8 eV and 400.5 eV, which can be attributed to N-Ti-O and C-N, respectively^39^. The presence of C-N indicates that chitin is attached to the carbon fiber surface. In Fig. 4b O 1 s XPS spectrum, there are three main peaks centered on 284.66 eV, 285.16 eV, and 286.15 eV that can be attributed to sp^3^ hybrid carbon (C-C) and sp^2^ hybrid carbon (C = C) 1 s electrons, which form a C-OH bond^40^. The sample has two peaks at 458.65 eV and 464.45 eV corresponding to the Ti^4+^ atoms in TiO_2_ (as shown in Fig. 4c). For the O 1 s electrons (Fig. 4d), the sample peak at 532.83 eV corresponds to the surface of TiO_2_. Hydroxyl O has another strong peak at 529.6 eV, which is assigned to the oxygen bonded to Ti^41^.

**Figure 5:**
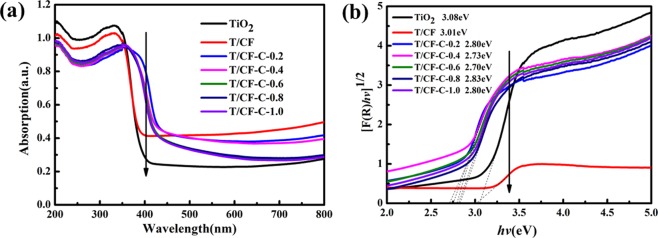
The UV–Vis DRS spectra (**a**) and band gaps of chitin, T/CF, and so-prepared composites (**b**).

Optical properties of T/CF samples were investigated via UV-Vis diffuse reflectance spectroscopy (DRS, Fig. 5a). Moreover, the band gaps of TiO_2_ and the prepared sample were calculated and listed in Fig. 5b. As shown in Fig. 5a, by comparing he absorption starting points of the prepared T/CF composites and pure TiO_2_ at 420 nm in the visible light region under the same operating conditions, we can notice that the absorption extends and the optical absorption undergoes a red shift when chitin has been added. which may be related to the photosensitivity of chitin, isomerizing under photoirradiation and enhancing the visible light response. Therefore, we conclude that the coordination among TiO_2_, carbon fiber, and chitin molecules may be the main reason for the improvement of the photocatalytic activity of the catalyst.

**Figure 6:**
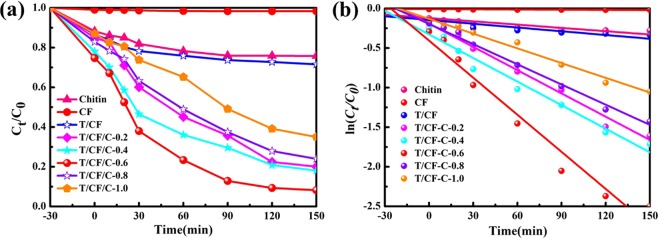
Photocatalytic degradation of RhB with T/CF composites as photocatalysts under visible light (**a**) and linear transform of the kinetic curves of the RhB degradation (**b**).

The photocatalytic activity of the T/CF composites was investigated by measuring the photodegradation of RhB in aqueous solution (50 mg/L) under visible light irradiation (Fig. 6a). For comparison, the photocatalytic activities of pure chitin, carbon fiber, and TiO_2_ were also determined under the same operating conditions. It can be seen from Fig. 6a that the concentration of the control RhB solution remains unchanged upon direct irradiation, while the strength of the RhB solution with chitin is reduced by about 20% at a reaction time of 180 minutes, which can be attributed to the strong adsorption of RhB on porous chitin materials^42^. The T/CF-C-0.6 sample exhibits the highest adsorption capacity and the degradation rate for RhB, which is almost 4 times higher than that of pure TiO_2_. However, due to the shielding effect, an excessive amount of adsorption dye will reduce its activity. The higher performance of the composites can be attributed to the synergistic effect of the TiO_2_ and chitin crystal phases, the high surface area, and the porous structure of the composites. The TiO_2_ nanoparticles exhibit excellent photocatalytic efficiency by transferring photoelectrons from the bulk to the surface and reducing the recombination rate of hole-electron pairs. The kinetic data of RhB photodegradation were obtained by the first-order reaction model as follows [Eq. (1)],

1 $$\mathrm{ln}(\frac{C}{{C}_{0}})=-\,{K}_{obs}t$$

**Figure 7:**
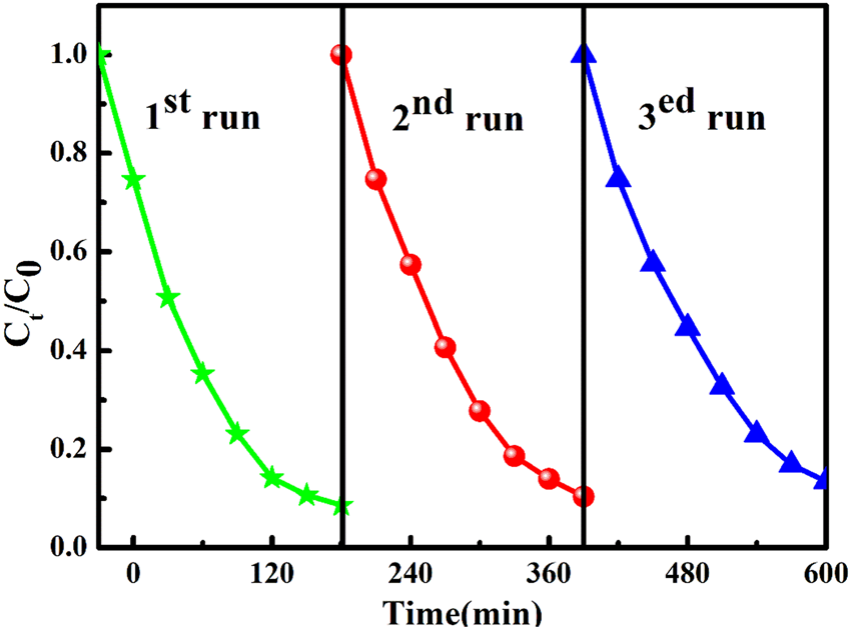
Reusability test of the T/CF-C-0.6 sample under visible light.

**Figure 8:**
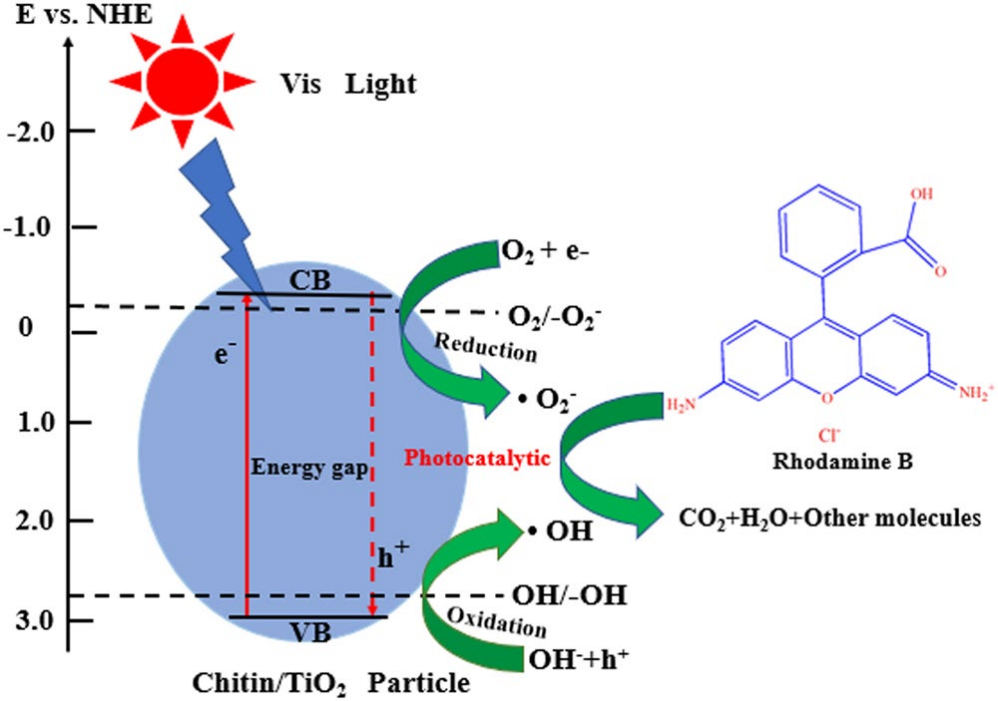
Proposed mechanisms for the RhB degradation of the so-prepared composite photocatalysts under visible light.

Where *k*_*obs*_ is the apparent first-order rate constant (min^−1^), *C*_0_ is the initial RhB concentration (mol/L), and *C* is the RhB concentration (mol/L) in the aqueous solution at time t. The kinetic curve of degradation of RhB as a function of the T/CF composites is displayed in Fig. 6b. All photocatalysts are suitable for pseudo first-order models. Our kinetic results show that the *k*_*obs*_ values of chitin, T/CF,T/CF-C-0.2, T/CF-C-0.4, T/CF-C-0.6, T/CF-C-0.8, T/CF-C-1.0, and carbon fiber are 0.0035 min^−1^, 0.01021 min^−1^, 0.01565 min^−1^, 0.01888 min^−1^, 0.02539 min^−1^, 0.01459 min^−1^, 0.01209 min^−1^, >10^-4^ min^−1^, respectively. Therefore, the T/CF-C-0.6 sample obtained the best photocatalytic efficiency, which is 2.5 times higher than that of T/CF. Indeed, it is known that a photocatalyst having a relatively high specific surface area is advantageous for increasing catalytic activity. The T/CF-C-0.6 sample has the highest specific surface area and the highest catalytic activity, indicating that small grain size and small band gap width should be the key factors for improving the photocatalytic activity. This is consistent with our previous BET results shown in Table 1. In this case, the excellent photocatalytic activity can be attributed to the direct adsorption of RhB on TiO_2_ and chitin as well as the photodegradation of the adsorbed dye on the TiO_2_ catalytic surface under visible light irradiation. The photocatalytic degradation can be enhanced by increasing the amount of dye adsorbed on the adsorbent; therefore, the action of chitin is indispensable in photocatalytic degradation because of its porous structure and high surface area, providing a more easily available adsorption site. The stability of TiO_2_ and chitin loaded on carbon fiber was evaluated by performing a cyclic experiment of RhB photocatalytic degradation. After each test, the used TiO_2_/carbon fiber composites were dried at 80 °C after being washed by deionized water. The reusability results of the T/CF-C-0.6 sample are reported in Fig. 7. Indeed, the RhB degradation rate of T/CF composites has not decreased significantly after three reaction cycles, which implies that the so-synthesized T/CF photocatalyst exhibits good reusability. These results indicate that the nanoparticles are stable on the carbon fiber surface, while a strong interaction exists between these two materials, which will not peel off as the reaction progresses. Furthermore, the photocatalytic degradation functions of the modified T/CF composites are reproducible. Figure 8 illustrates the process of RhB degradation caused by T/CF composites. Upon irradiation, electrons are directly transferred from the valence band into the conduction band of TiO_2_, while the molecular oxygen (O_2_) is reduced to superoxide radicals (O●^−2^). The hole electrons then react with water to form hydroxyl radicals (OH●)^43^. Subsequently, under visible light irradiation, these free radicals are responsible for the degradation of the adsorbed RhB molecules into inorganic ions and small molecules, such as water and carbon dioxide.

## Conclusions

In summary, chitin-modified TiO_2_ nanoparticles with excellent photocatalytic properties in the region of visible light were synthesized successfully on the surface of carbon fiber using the hydrothermal method. Acting as a modifier and adsorbent, the addition of chitin not only broadened the photoresponse range but also improved the photocatalytic performance of TiO_2_. Indeed, photocatalytic analysis results showed that modified T/CF composites have better adsorption and photocatalytic properties than single chitin and TiO_2_ nanoparticles under visible light. The apparent rate constant of degradation of the T/CF-C-0.6 sample was approximately 2.25 times higher than that of T/CF. In addition, T/CF-C-0.6 sample showed good stabilization of catalysis after three cycles of the test. The present work provides an effective route to enhance the catalysis performance of TiO_2_ in the visible light region and extend their application to various fields, such as solar energy conversion and self-cleaning, especially photocatalytic degradation of organic pollutant in wastewater.

## Methods

Tetrabutyl titanate (TBT) purchased from Sinopharm Chemical Reagent Ltd. (China) was chosen as the precursor of TiO_2_. Glacial acetic acid (CH_3_COOH, 99.8%) and ethanol (C_2_H_5_OH, 99.7%) purchased from Beijing Chemical Reagent Ltd. (China) were used as the catalyst and solvent, respectively. Chitin was purchased from Yuhuan Ocean Biochemistry Co. Ltd. (China). Carbon fiber (CF) was purchased from Nantong Senyou carbon fiber Co. Ltd. All reagents were of analytical grade and used without further purification. TiO_2_/carbon fiber composites were prepared via hydrothermal method. During the classical synthesis process, 2.5 mL of TBT was slowly added dropwise to 15 mL of absolute ethanol, to which 5 mL of glacial acetic acid is added and then stirred for 3 hours. 0.2 wt%, 0.4 wt%, 0.6 wt%, 0.8 wt%, and 1.0 wt% of chitin were thereafter added to the mixed solution as a modifier. After further stirring for another hour, the solution was then transferred to a Teflon stainless-steel autoclave with a total volume of 25 ml. With this done, acetone-treated carbon fiber was immersed in the solution. The hydrothermal synthesis was carried out by placing the solution in an oven set at 180 °C for 24 hours. When the autoclave has cooled to room temperature, the carbon fiber was taken out and ultrasonically washed in an aqueous ethanol solution for 2 minutes. It was then dried in an oven set at 150 °C for 8 hours. Finally, the photocatalytic material of pure TiO_2_/carbon fiber as well chitin-modified TiO_2_/carbon fiber composites are obtained, labeled as T/CF, T/CF-C-0.2, T/CF-C-0.4, T/CF-C-0.6, T/CF-C-0.8, T/CF-C-1.0, depending on different amount of modifier of chitin: 0.2 wt%, 0.4 wt%, 0.6 wt%, 0.8 wt%, and 1.0 wt%, respectively.

The crystal phases were characterized via a powder X-ray diffraction (XRD, TD-3500X, Dandong Tongda Technology Co., Ltd.), operating at 40 kV and 40 mA with a Cu Kα irradiation. The surface morphologies were observed via scanning electron microscopy (SEM, Carl Zeiss, SIGMA 500, Germany). The UV–vis diffuse reflectance spectra were recorded by UV2550 UV/vis spectrophotometer (Shimadzu Corporation Japan) with the scanning range set at 200–800 nm, using BaSO_4_ as the reference. X-ray photoelectron spectra (XPS) were recorded with a Kratos AXIS Ultra DLD XPS instrument equipped with an Al Kα source at 10^−9^ Torr. The specific surface and pore size distribution of the sample were measured by the US Mike TRISTAR II 3020 M for nitrogen adsorption/desorption. The photocatalytic activity of these prepared T/CF composites was measured by the time evolution of photodegradation of rhodamine B (RhB) under a 500 W Xenon lamp with a 420 nm cut-off filter. During the process of testing photocatalytic performance, the amount of photocatalyst should be consistent. In a typical procedure, 50 mg of the TiO_2_/carbon fiber composites were added into an aqueous solution of RhB (50 mL, 50 mg/L). Then, the mixture stirred in the dark for 30 min at room temperature to achieve the adsorption-desorption equilibrium. At the defined time intervals, 3 mL of the solution was taken from the reactor. The concentration of RhB in the suspension was monitored using the UV–vis spectrophotometer at the intensity of the absorption peak of 554 nm; the photocatalytic activities of the samples were calculated from the decrease amount of RhB.
